# Elevated Sperm DNA Damage in IVF–ICSI Treatments Is Not Related to Pregnancy Complications and Adverse Neonatal Outcomes

**DOI:** 10.3390/jcm12216802

**Published:** 2023-10-27

**Authors:** Irene Hervás, Rocio Rivera-Egea, Alberto Pacheco, Maria Gil Julia, Ana Navarro-Gomezlechon, Laura Mossetti, Nicolás Garrido

**Affiliations:** 1 IVIRMA Global Research Alliance, IVIRMA Rome, Via Federico Calabresi, 11, 00169 Rome, Italy; irene.hervas@ivirma.com (I.H.); laura.mossetti@ivirma.com (L.M.); 2 IVIRMA Global Research Alliance, IVIRMA Valencia, Andrology Laboratory and Sperm Bank, Plaza de la Policía Local 3, 46015 Valencia, Spain; rocio.rivera@ivirma.com; 3 IVIRMA Global Research Alliance, IVIRMA Madrid, Andrology Laboratory and Sperm Bank, Av. del Talgo 68-70, 28023 Madrid, Spain; alberto.pacheco@ivirma.com; 4 Faculty of Health Sciences, Alfonso X el Sabio University, Avda. de la Universidad, 1, Villanueva de la Cañada, 28691 Madrid, Spain; 5 IVIRMA Global Research Alliance, IVI Foundation, Instituto de Investigación Sanitaria La Fe (IIS La Fe), Avenida Fernando Abril Martorell, 106—Torre A, Planta 1ª, 46026 Valencia, Spain; maria.gil@ivirma.com (M.G.J.); ana.navarro@ivirma.com (A.N.-G.)

**Keywords:** SDF, DNA damage, neonatal outcomes, oocyte donation, ICSI, IVF

## Abstract

This multicenter retrospective cohort study assesses the effect of high paternal DNA fragmentation on the well-being of the woman during pregnancy and the health of the newborn delivered. It was performed with clinical data from 488 couples who had a delivery of at least one newborn between January 2000 and March 2019 (243 used autologous oocytes and 245 utilized donated oocytes). Couples were categorized according to sperm DNA fragmentation (SDF) level as ≤15% or >15%, measured by TUNEL assay. Pregnancy, delivery, and neonatal outcomes were assessed. In singleton pregnancies from autologous cycles, a higher but non-significant incidence of pre-eclampsia, threatened preterm labor, and premature rupture of membranes was found in pregnant women from the >15%SDF group. Additionally, a higher proportion of children were born with low birth weight, although the difference was not statistically significant. After adjusting for potential confounders, these couples had lower odds of having a female neonate (AOR = 0.35 (0.1–0.9), *p* = 0.04). Regarding couples using donor’s oocytes, pregnancy and neonatal outcomes were comparable between groups, although the incidence of induced vaginal labor was significantly higher in the >15% SDF group (OR = 7.4 (1.2–46.7), *p* = 0.02). Adjusted analysis revealed no significant association of elevated SDF with adverse events. In multiple deliveries from cycles using both types of oocytes, the obstetric and neonatal outcomes were found to be similar between groups. In conclusion, the presence of an elevated SDF does not contribute to the occurrence of clinically relevant adverse maternal events during pregnancies, nor does it increase the risk of worse neonatal outcomes in newborns. Nevertheless, a higher SDF seems to be related to a higher ratio of male livebirths.

## 1. Introduction

The demand for assisted reproductive techniques (ART) is increasing every year throughout the world. This is due to the socioeconomic changes affecting each new generation, which represent the main cause of delayed childbearing [1]. It is estimated that up to 6% of children born in Europe were conceived by ART, with the number of pregnancies resulting from ART having surpassed one million per year since 2018 [2]. Similarly, the number of cycles initiated in the USA continues to increase; an estimated 1.5% of children have been born because of ART since 2006 [3]. 

Given the increasing demand for ART, in recent years, concerns have arisen about the short- and long-term safety of these treatments for both mothers and offspring. It has been noted that the application of ART is associated with a more significant number of obstetric complications during pregnancy, such as placental abnormalities or hypertensive disorders (pre-eclampsia) [4], and with the need for interventions, such as caesarean section [5,6]. Likewise, pregnancies resulting from ART have been historically associated with an increased risk of preterm delivery in singleton pregnancies [7,8] and low birth weight [9,10] compared to pregnancies conceived naturally. A large population-based cohort study comparing births between 2004 and 2016 in the United States [11] found that singleton infants born from infertility treatments using autologous oocytes (78,362 cases) had a higher risk of non-chromosomal defects, cardiovascular and musculoskeletal defects, and major defects than naturally conceived children (1,037,757 cases). Moreover, this risk is increased with treatments involving the ICSI technique, and more so with male infertility (18%, 30%, and 42%, respectively). Moreover, the latest systematic review [10], which compared more than 180,000 singleton pregnancies from IVF/ICSI cycles with newborns spontaneously conceived, showed that ART children have more compromised perinatal outcomes (higher rates of preterm birth, low and very low birth weight, increased risk of small gestational age) after adjusted risks.

However, it is not well understood why ART causes these adverse events in mothers and children. The different processes that currently involve infertility treatments have been studied separately. The application of different IVF techniques, such as the freezing and thawing of embryos, did not entail significant differences in gestational age, newborn weight, or neonatal morbidity when compared to pregnancies of the same women when donated oocytes were used [12,13]. Moreover, no adverse effects were found in children born from pregnancies obtained from embryos cultured using time-lapse technology [14], and a German historical cohort study after 21 years of follow-up of ART-conceived children found no increased risk of cancer compared to naturally conceived children [15]. It is not entirely clear that the infertility treatment causes these events, but rather that they may be associated with the patient’s infertility condition or their advanced maternal age, as occurs in ART populations [16,17]. 

Ensuring the safety of the ART applied must be a prerequisite to guarantee the achievement of healthy progeny and preserve the well-being of the pregnant mother. To this end, it is essential to seek excellence in the work protocols applied; however, it remains to be determined whether the adverse effect is produced by the laboratory processes proper to ART or by the quality of the gametes involved. In this regard, paternal DNA damage has been extensively studied in regard to its relationship with reproductive outcomes. Several studies to date have shown that damaged paternal chromatin can impair fertility potential in natural and ART conceptions [18]. Moreover, it has been reported to be more common in males who undergo ART [19,20], but is associated with both abnormal [21,22] and normal seminal parameters [23,24].

Currently, the deleterious effect of sperm DNA fragmentation (SDF) on the likelihood of pregnancy in these couples remains controversial. Some authors state that SDF impairs embryo quality [25,26], significantly reducing the pregnancy rate in favour of an increased probability of miscarriage [27,28,29,30]. Others, however, did not find that fragmented paternal chromatin leads to poor reproductive outcomes after IVF or ICSI treatments [31,32,33]. Conversely, very few studies have evaluated its effect beyond pregnancy itself, and whether it increases the risk of adverse neonatal outcomes or impairs mother’s well-being along gestation remains unexplored. 

It is well known that sperm with fragmented DNA can fertilize an oocyte and give rise to an embryo. The oocyte contains the repair machinery necessary to restore the integrity of the paternal genome [34] and produce a competent embryo capable of implanting and giving rise to living offspring. However, it is unclear in these cases if the repair is complete or if it is performed in the correct pattern. The extent of the breaks may be in regulatory areas and coding genes, thus affecting epigenetic processes. Additionally, if DNA breaks or if restoration is not well complete, chromosomal aberrations may appear, with future consequences in the offspring. 

The first study to assess the effect of SDF on progeny was conducted in 2012 [35]. The study found no statistically significant differences in birth weight and gestation length between 131 singleton pregnancies in couples with different degrees of SDF (20%, 30%, 40%, and >50%, measured by SCSA (Sperm Chromatin Structure Assay)) after IVF and ICSI cycles. However, the number of cases was small, so the statistical power is low. In another study recently published by Chen et al. in 2020 [36], they retrospectively evaluated 713 ICSI cycles with one delivery after 1139 embryo transfers (ETs) in couples, divided according to different degrees of SDF (≤15%, 15–30%, and >30% by SCSA). They found that high SDF did not increase the risk of perinatal and neonatal deaths, and neither were the neonatal variables of gestational age, prematurity, sex, and birth weight affected between the groups. However, despite the retrospective nature, they failed to perform an adjusted analysis of the results obtained for controlling the possible effects of confounding variables. On the other hand, the effects on offspring after oocyte injection with SDF were assessed in a mouse model. They observed an increased risk of long-term effects in these born mice related to premature aging, abnormal growth, unknown epigenetic changes, and the appearance of tumours [37]. 

To date, only these two studies have been conducted, but since SDF is a common problem in many men who conceive, further analysis is needed to better assess its impact. In addition, this issue has been analysed only in couples who used their oocytes, with no information on this question in donated oocytes, a very common treatment nowadays. Thus, due to the small amount of available information on the subject, additional evaluation is needed to ensure that IVF treatments are safe for the mother’s well-being during pregnancy and for the ART children. In the present study, the aim is to evaluate the risk of the women to suffer any adverse obstetric event during pregnancy and the health of the newborns in couples with SDF in their IVF/ICSI treatment using both own and donated oocytes.

## 2. Materials and Methods

### 2.1. Study Population

In this multicentre retrospective study, obstetric and perinatal data were gathered from women who became pregnant after undergoing an IVF/ICSI cycle in our Spanish centres between January 2000 and March 2019 and whose partner had a sperm DNA fragmentation test. Data from cycles performed with both own and donated oocytes were included but analysed separately. In all cases, each woman had a delivery with at least one live birth. The indications for SDF testing were previously indicated [32,33]. The data exported from our database were analysed anonymously. 

### 2.2. IVF Laboratory Procedures

All patients using their oocytes underwent controlled ovarian stimulation with a GnRH-agonist or -antagonist protocol [38,39]. When three follicles had reached a diameter >18 mm, hCG was administered and transvaginal oocyte retrieval was scheduled 34–36 h later. Oocyte donors were young (18–35 years old) and healthy anonymous women volunteers. The donors were physically and genetically matched with the recipients. All donors were screened for sexually transmitted diseases and were in good physical and mental health [40]. The ovarian stimulation protocol and the successive oocyte retrieval were performed following previously described protocols [41]. 

Mature oocytes were inseminated by conventional IVF or by ICSI techniques [39] as described. For cases in which previously cryopreserved oocytes were used, the vitrification and warming procedure used was previously reported [38]. Inseminated oocytes were cultured under controlled conditions (6% CO_2_, 5% O_2_, 37 °C) until day 5 or 6 of embryonic development. At that time, the appropriate embryos were transferred (at D3 or D5/6) or vitrified according to the requirements of each patient’s cycle. For embryo transfer, patients could undergo an artificial endometrial preparation following the already published protocols [42], or according to their natural cycle if required, until reaching an adequate endometrial thickness. All patients received luteal phase support with micronized vaginal progesterone until at least 8 weeks of pregnancy.

### 2.3. Sperm Analysis

Sperm samples were collected into a sterile recipient by masturbation after 3–5 days of sexual abstinence. They were evaluated for volume, sperm count, motility, and morphology according to World Health Organization criteria [43]. If necessary, the semen sample was frozen for later use [44]. Samples were prepared for insemination by density gradients [39] or by swim-up [45]. 

Sperm DNA fragmentation analysis was performed using Terminal deoxynucleotidyl transferase biotin dUTP nick end labelling (TUNEL) assay following the manufacturer’s instructions (In situ Cell Death Detection Kit by Roche Diagnostics, Barcelona, Spain). The analysis was performed on fresh semen samples at IVI Madrid, as previously published [39,46], on a minimum of 10,000 sperm cells. The proportion of sperm with fragmented DNA was measured using a FACScan (Becton Dickinson, Franklin Lakes, NJ, USA) cytometer until 2015, and since then, using a MACSQuant (Miltenyi Biotec, Bergisch Gladbach, Germany) cytometer.

### 2.4. Outcomes

Clinical data related to obstetrics, delivery and perinatal aspects were acquired by standard maternal survey administered by medical staff through telephone calls or emails with patients.

The obstetric variables measured were gestational diabetes, anaemia (haemoglobin ≤ 11 g/dL), and pre-eclampsia (defined as the presence of hypertension and proteinuria), and those related to risks of pregnancy loss (threatened preterm delivery, first-, second-, and third-trimester bleeding, and premature rupture of membranes (PROM ≤ 37 weeks)). Delivery outcomes were weeks at delivery, caesarean section, induced vaginal labour, puerperal problems, and preterm (≤37 weeks) and very preterm (≤34 weeks) births. Neonatal outcome measures were gender, birth weight (referring to low (≤2500 g) and very low weight (≤1500 g)), birth height, and neonatal morbidity (Apgar score at 1, 5, and 10 min, and admission to the neonatal intensive care unit). 

### 2.5. Statistical Analysis

The study population was divided into two groups based on the level of SDF greater or less than 15% to perform the analysis. To compare the variables, the origin of the oocyte (autologous or donated oocytes) and whether the pregnancy was singleton or twin were also considered. Continuous and categorical variables were expressed as means or proportions with their respective 95% confidence interval (95% CI). Continuous variables were compared using Student’s *t*-test, whereas categorical variables were compared with a Chi-squared test or a Fisher’s exact test, where appropriate. A *p*-value < 0.05 was considered statistically significant. In addition, logistic regression analysis was performed on perinatal data for preterm birth ≤37 weeks, very preterm birth ≤32 weeks, female neonates, and birth weight ≤2500 g, according to the degree of high SDF (>15%). To reduce the risk of clinical bias, the following confounding variables were included in the multivariate logistic regression analysis: female and male age, female body mass index (BMI), day of embryo transfer (D3 or D5), and endometrial thickness. Statistical analysis was performed using R software (4.02 version) and a *p*-value < 0.05 was considered statistically significant. 

## 3. Results

### 3.1. Baseline Demographics and Characteristics of the Study Population

This study included a total of 488 couples who had a delivery with at least one newborn and presented sperm DNA fragmentation analysis in their treatment. A total of 243 couples used their own oocytes (212 were singleton and 31 were multiple) and 245 couples used donated eggs (212 were singleton and 33 were multiple). Both types of treatments were assessed separately. Couples were counted only once until they achieved full-term pregnancy with the birth of one or more children. In addition, pregnancies with more than one child were analysed independently from singleton deliveries. 

The baseline characteristics of each study group in single deliveries are described in Table 1, with no differences between them in terms of maternal and paternal age, patient BMI, controlled ovarian stimulation outcomes, and the number of embryos transferred per patient. Supplementary Material Table S1 present the epidemiological characteristics of patients with multiple pregnancies using their own and donated oocytes.

### 3.2. Outcomes of Singleton Deliveries of Couples using Autologous Oocytes

Table 2 presents the pregnancy, delivery, and neonatal outcomes. In univariant analysis, the variables examined showed a comparable occurrence between both groups, although the >15% SDF group had a higher prevalence of pre-eclampsia, threatened preterm labour, and PROM. Regarding delivery outcomes, no differences in gestational age or in the proportion of deliveries that ended in caesarean section were observed between groups; additionally, a higher proportion of children born below 37 weeks was noted in the ≤15% SDF group but was not statistically significant. However, the proportion of very preterm births was similar between the two groups. 

Regarding neonatal outcomes, a lower but non-significant proportion of babies were born with low weight (7.1% (3.1–13.6) vs. 11.8% (1.2–36.4)) and very low weight (0.9% (0.0–4.9) vs. 5.9% (0.2–28.7)) in couples with an SDF ≤ 15% compared to couples with SDF > 15%. No statistical differences were observed in the other neonatal outcomes, but twice as many males as female neonates were observed in the <15% SDF group compared to the other group. 

### 3.3. Outcomes of Singleton Deliveries of Couples using Donated Oocytes

Regarding couples subjected to an oocyte donation program, data are shown in Table 3. Women in the >15% SDF group had a higher occurrence of gestational diabetes, 12.5% vs. 2.9% (≤15% SDF), bleeding during pregnancy, 25.0% vs. 13.2% (≤15% SDF), and higher risk of early rupture of membranes, 12.5% vs. 2.2% (≤15% SDF), though the difference was not statistically significant. Concerning delivery outcomes, the weeks at delivery and the performance of caesarean sections in childbirth were comparable between the groups. However, a significant difference was found in the incidence of induced vaginal labor in women from the >15% SDF group, 44.4% (13.7–78.8), compared to those in the ≤15% SDF group, 9.4% (3.5–19.3). A higher but not statistically significant number of children were born before 37 and 34 weeks of gestation to couples with high SDF. There were no significant differences in neonatal outcomes between the couples, though a higher proportion of the children were born with low weight in the >15% SDF group, while there were no reported cases with very low weight. The remaining parameters were comparable between the two groups.

### 3.4. Obstetric and Neonatal Outcomes in Multiple Deliveries

In pregnancies of couples using autologous oocytes (Supplementary Material Table S2), no adverse pregnancy events were found among the groups. However, a high but not significant rate of preterm births and low birth weight were found in the ≤15% group. Additionally, couples from the high SDF group had a greater proportion of male neonates (non-significant) than couples with low DNA damage. 

Supplementary Material Table S3 shown the outcomes of deliveries from cycles using donated oocytes. Few adverse events were reported from the high SDF group, although a higher but not statistically significant proportion of children born at less than 37 weeks was found compared to the lower SDF group. Similar neonatal outcomes were observed among both groups.

### 3.5. Multivariable Analysis

A logistic regression analysis adjusted to potential confounding variables was performed in neonatal outcomes for the presence of an SDF higher than 15%. The results are shown in Table 4. After adjusting, lower although non-significant odds of having a preterm (AOR = 0.31 (0.0–0.2)) or very preterm birth (AOR = 0.80 (0.0–0.6)) and a small birth weight (AOR = 1.6 (0.2–9.4) was found in the group with autologous oocytes. However, a statistically significant relationship was noted in the adjusted odds of having a female newborn (AOR = 0.35 (0.1–0.9) (*p* = 0.04). Whereas, in couples using donated oocytes, increased odds were found for the variables preterm and very preterm birth (AOR = 1.76 (0.5–5.9) and AOR = 2.6 (0.4–14.3), respectively), and for birth weight ≤2500 g (AOR = 2.42 (0.3–13.2), always in a non-significant manner. However, to the contrary, in this group, the high SDF did not significantly affect the odds of having a female newborn (AOR: 0.70 (0.3–1.6) (*p* = 0.39).

## 4. Discussion

This retrospective study aimed to evaluate the effect of an elevated paternal SDF in the IVF/ICSI cycles on obstetrical and perinatal outcomes in deliveries from couples using both autologous and donated oocytes. In this case, single deliveries were analysed separately from multiple ones. We found that the presence of high paternal chromatin damage does not appear to have any medically relevant effect on the well-being of the pregnant woman or on the health of the children born following both types of treatment. Additionally, we observed that high SDF impacts the sex ratio of the offspring, favouring the birth of males in couples that underwent a cycle with their own oocytes, whereas this influence was not seen in cases of oocyte donation. The same effect was observed in multiple deliveries. For us, this is the largest study ever conducted, as it examines the effect of paternal DNA fragmentation on both the health of the pregnant woman and the child born after ART treatment, considering both types of oocytes, own and donated.

In assisted reproduction, a healthy newborn is the most essential objective for patients seeking parenthood and for clinicians. A huge systematic review of 180,000 singletons born [10] and 60,210 multiple births [9] after ART showed an increased risk of pre-term birth, small gestational age, and low birth weight compared to naturally conceived children. In addition, altered foetal and prenatal development could determine immaturity in different organs and affect reproductive adult health, as shown by [47]. Other authors, however, did not find such a negative association [11,14,15]. Therefore, there is growing evidence of information regarding the safety of assisted reproductive technologies and the health of children born through them. Indeed, several studies have evaluated how ART-associated processes such as ovarian stimulation [48,49,50], embryo culture [14,48], and cryopreservation [12,13,51], as well as the type of preparation for ET [52,53] might influence obstetric and perinatal outcomes. However, there is a lack of information on the involvement of SDF at the time of conception beyond the usual clinical outcomes.

Thus far, the assessment of sperm DNA fragmentation has been added to the arsenal of clinical tests performed on couples having difficulty conceiving. Nevertheless, due to the disparity in SDF assays and the heterogeneous populations studied, the impact on clinical outcomes remains a matter of debate. Likewise, it is recognized that the degree of expansion of breaks in the paternal chromatin is variable. Consequently, different genes including coding and non-coding sequences related to human development may be compromised. Moreover, if the repair machinery of the oocyte is insufficient or erroneous, DNA breaks can lead to mutations inherited by the offspring, as was formerly demonstrated with an animal model [37]. Hence, it is important to assess how SDF may be related to the occurrence of adverse medical events in infants.

The first study assessing the impact of spermatozoa DNA damage on birth outcome was performed by Bungum et al., 2012 [35], who evaluated the gestational age and birth weight of 131 singletons born after conception by IVF and ICSI. The population was divided according to the level of SDF into 20, 30, 40, and 50% fragmentation groups. They concluded that high values of SDF were not associated with birth weight and gestational length. More recently, in a second published study [36], the comparison of 713 singleton deliveries from ICSI cycles with different levels of SDF concluded no negative effects on neonatal outcomes. In this case, high SDF also did not significantly increase the risk of low birth weight or prematurity. Nor did they observe a higher number of congenital defects or perinatal deaths. Therefore, there is an important lack of related information, which requires evaluation. 

In our study, SDF was high from 15% fragmentation onwards. When singleton deliveries from autologous cycles were assessed, even though the differences were not statistically significant, a paternal DNA fragmentation >15% seemed to favour the incidence of pre-eclampsia and the threat of preterm labour. The gestational week of delivery was comparable between both groups. Commonly, higher rates of preterm births are associated with the application of IVF and ICSI treatments [6,7]. In this study, we observed that the delivery of these children was earlier than 40 weeks, but in no case was there a high rate of preterm and very preterm. Therefore, it does not appear that elevated SDF has an added effect on gestational age compared to ART itself, as confirmed by the multivariate analysis performed. 

Regarding neonatal outcomes, children born to couples with high SDF had results comparable to those of the other group, with small and non-significant differences. Nor was a greater number of adverse events observed in these newborns. Though an increased number of newborns with a low birth weight relative to the standard were born in this group, the difference was not statistically significant. This finding is in line with the two previous studies [35,36] and was confirmed with our adjusted analysis. Notwithstanding, we noted a higher but not significant ratio of male than female neonates in the group with an SDF >15%, which was also found in Chen’s study results [36]. The subsequent adjusted analysis for variables that could have influenced the result confirmed significantly lower odds of having a female neonate in couples with higher SDF, thereby losing the male–female balance. The reason for this phenomenon needs to be studied with larger samples. We hypothesize that differences in the size of the sex chromosomes may be reflected in an increased number of DNA breaks in the X chromosome than in the Y chromosome, or that damage restoration is not as successful as expected in the X one, resulting in impaired embryo development that eventually does not give rise to live birth.

On the other hand, the involvement of SDF in deliveries and adverse neonatal outcomes from an oocyte donation program has not yet been evaluated, so this is the first study to assess it. The virtue of this type of treatment is that it allows us to standardize the female factor using a healthy young oocyte. For these couples, a higher incidence of gestational diabetes, pregnancy bleeding, and PROM was observed in women in the >15% SDF group but always these differences were not statistically significant. Additionally, we found comparable weeks at delivery between both groups, although an increased incidence of preterm birth was observed in the group with elevated SDF. After the proper adjustment, we observed that having an elevated SDF increases the odds of preterm deliveries, but this relationship was not statistically significant. Interestingly, a greater proportion of women needed induced vaginal labour at delivery, but we consider this finding of limited clinical relevance due to it being a gynaecological common practice, and because we do not know if it was at the patient’s request or for medical reasons.

Nevertheless, we cannot discern whether these incidents are, in turn, related to the egg donation or to the initial presence of paternal DNA damage, as pregnancies of women with babies from egg donation have been reported to be associated with a higher incidence of medically adverse events than those born from natural conceptions, and it is thought that the difference could be mainly related to immunological causes [4,50,54]. Moreover, no increased incidence of serious adverse clinical events was observed among the neonates from couples with elevated SDF. Meanwhile, a double-higher incidence of babies with low weight was noted in the >15% SDF group; however, even though the adjusted analysis revealed increased odds of this phenomenon in these couples, it was not a statistically significant difference. Nonetheless, we may not have been able to detect this association because of the low number of cases. Additional studies are needed to confirm this finding. Finally, in this population, the sex ratio of newborns was comparable between both groups before and after adjusting for confounding variables. The reason that the disturbance of sex ratio does not occur in oocyte donation cycles may be due to the quality of the cytoplasm of the young oocyte, which has a piece of more efficient repair machinery than that of an older oocyte [34], correcting with the same efficacy the chromatin damage in both chromosomes.

Concerning outcomes from multiple deliveries, it is more difficult to assess the effect of paternal DNA fragmentation on its own, as multiple pregnancies present more risks than singleton ones, as is known [51,55]. In couples that used autologous eggs, no higher incidence of adverse events was observed in mothers and infants born from couples with a high SDF, but on the contrary, a higher proportion of infants were born preterm before 37 weeks and with low birth weight in the <15% SDF group. In addition, they experienced a higher number of NICU admissions. Curiously, once again, a higher rate of male neonates, although not significantly so, was observed in the group with high DNA damage. Furthermore, we cannot effectively explore the impact of >15% SDF on multiple deliveries in couples using donated oocytes due to the lack of available data and the small study population evaluated. Even so, the mean delivery week and neonatal outcomes were comparable between both groups; only a higher but not significant proportion of neonates was born prematurely and with low weight in >15% SDF group, which is a normal event in multiple ART pregnancies [9]. 

Our study, however, presents some limitations that should be considered. Firstly, its retrospective design and the time-related bias due to the long period considered should be noted. However, this study design allows for the evaluation of a high amount of data, so, consequently, the statistical analysis was adjusted to account for critical confounding factors. Secondly, the lack of follow-up in some pregnancies or the patient not adequately completing the survey leads to the loss of valuable information regarding outcomes and possible adverse events that may have happened, which could underestimate the effect.

A major change that has occurred in recent years is the increased number of replaced embryos at embryo transfer, which is directly related to multiple pregnancies and their risks. Therefore, we considered it essential to separate the neonatal and obstetrical results between single and multiple births, as well as the origin of the oocyte, for possible involvement in the clinical outcomes when the oocyte has a different genetic origin from the mother. Therefore, the low study population of multiple births hinders firm conclusions.

To end, this study offers new evidence of the obstetric and neonatal outcomes of couples who performed their cycles with sperm DNA damage in both homologous and heterologous treatments. Despite the number of cases evaluated, it is necessary to enlarge the sample to corroborate the findings, especially those related to the sex of the newborns and the birth weight in the case of couples with high SDF. Continued health surveillance of the offspring of these couples who resort to infertility treatments should be followed to monitor the long-term effects. 

## 5. Conclusions

In conclusion, SDF was not significantly associated with medically adverse events in pregnant women nor with worse neonatal outcomes in couples who undertook an IVF–ICSI treatment using both autologous and donors’ oocytes. In addition, the presence of sperm DNA fragmentation greater than 15% was associated with a higher chance of conceiving a male than a female in cycles with their oocytes, while this phenomenon was not observed in cycles with oocyte donation. Nevertheless, controlled prospective trials should be conducted to confirm these findings. 

## Figures and Tables

**Table 1:** Baseline demographics and characteristics of IVF cycles of couples using autologous and donated oocytes according to SDF value in single deliveries.

Variables	Autologous Oocytes					Donated Oocytes				
	≤15% SDF (95%CI)		>15% SDF (95%CI)		*p*-Value	≤15% SDF (95%CI)		>15% SDF (95%CI)		*p*-Value
Maternal age (years)	37.7 (114)	37.1–38.2	37.5 (25)	36.2–38.1	0.9	41.9 (136)	41.3–42.6	41.8 (16)	40.1–43.5	0.9
Female BMI (kg/m^2^)	22.3 (119)	21.7–22.9	22.4 (25)	21.4–23.5	0.9	23.1 (144)	22.5–23.7	22.2 (24)	21.1–23.2	0.1
Male age (years)	38.9 (175)	38.1–39.7	39.8 (37)	37.3–42.2	0.5	41.5 (181)	40.7–42.3	41.6 (31)	40.0–43.1	1.0
Male BMI (kg/m^2^)	22.2 (137)	21.6–22.7	22.8 (28)	21.7–23.8	0.3	23.2 (171)	22.7–23.7	22.5 (28)	21.4–23.6	0.3
SDF (%)	6 (163)	5.0–6.0	25 (36)	22.0–27.0	≤0.001	6.0 (171)	5.0–6.0	24.0 (28)	21.0–26.0	≤0.001
Previous miscarriages (≥1)	1.8 (40)	1.5–2.2	2.1 (11)	1.4–2.8	0.5	1.9 (64)	1.6–2.1	2.0 (4)	0.0–4.0	0.9
Parous women (≥1) %	14.2 (113)	8.3–22.0	8.0 (25)	1.0–26.0	0.5	11.1 (135)	6.4–17.7	6.3 (16)	0.2–30.2	1.0
Previous preterm deliveries (≤1) %	-	-	-	-	-	6.7 (15)	0.2–32.0	-	-	1.0
Previous medical disorders	14.2 (113)	8.3–22.0	16.0 (25)	4.5–36.1	0.8	16.3 (135)	10.5–23.6	6.3 (16)	0.2–30.2	0.5
Days of stimulation	10.6 (174)	10.3–10.9	10.7 (38)	10.2–11.1	0.8	10.7 (173)	10.5–10.9	10.2 (29)	9.7–10.8	0.1
FSH total dose (IU)	1650.6 (105)	1536.5–1764.7	1644.3 (22)	1339.5–1949.2	1.0	1690.5 (157)	1608.8–1772.1	1548.2 (27)	1347.9–1748.4	0.2
E2 on day of hCG (pg/mL)	2154.2 (166)	1960.3–2348.1	1943.6 (36)	1591.7–2295.5	0.3	2833.9 (164)	2549.2–3118.5	2587.5 (25)	1929.4–3245.7	0.5
P4 on the day of hCG (pg/mL)	0.6 (163)	0.5–0.7	0.6 (35)	0.5–0.7	0.5	0.6 (10)	0.4–0.9	2.7 (2)	2.1–3.4	0.1
Last endometrial thickness	9.8 (167)	9.6–10.1	9.7 (36)	8.9–10.5	0.8	9.2 (169)	8.9–9.5	9.2 (31)	8.7–9.7	1.0
Number of oocytes retrieved	11.1 (172)	10.1–12.0	12.0 (38)	10.2–13.7	0.4	20.6 (172)	19.3–21.8	21.7 (29)	18.3–25.1	0.5
Mean number of inseminations/patients	1.1 (163)	1.0–1.1	1.1 (36)	1.0–1.2	0.9	1.0 (171)	1.0–1.0	1.0 (28)	1.0–1.0	0.2
Embryos transferred/ patient	1.3 (163)	1.3–1.4	1.3 (36)	1.2–1.5	0.9	1.6 (171)	1.6–1.7	1.6 (28)	1.5–1.8	0.9
Day-3 ET (number)	12 (418)	-	8 (81)	-	-	34 (558)	-	6 (105)	-	-
Blastocyst ET (number)	406 (418)	-	73 (81)	-	-	524 (558)	-	99 (105)	-	-

Note: Values are expressed as mean or proportions (with its sample size). SDF—sperm DNA fragmentation; OR—odds ratio; CI—confidence interval; BMI—body mass index; FSH—follicle stimulating hormone; E2—estradiol; P4—progesterone; hCG—human chorionic gonadotropin; ET—embryo transfer.

**Table 2:** Obstetric and perinatal outcomes in singleton pregnancies of couples using their own oocytes, according to SDF value (*n* = 212).

Variables	≤15% SDF (95%CI)		>15% SDF (95%CI)		OR (95%CI)	*p*-Value
Pregnancy outcomes						
Gestational diabetes %	0.9 (112)	0.0–4.9	4 (25)	0.1–20.4	4.6 (0.1–365.1)	0.3
Anaemia (Hb ≤ 11 g/dL) %	1.8 (112)	0.2–6.3	-	-	-	1.0
Pre-eclampsia %	0.9 (111)	0.0–4.9	4.2 (24)	0.1–21.1	4.7 (0.1–377.5)	0.3
Threatened preterm labour %	0.9 (112)	0.0–4.9	4.0 (25)	0.1–20.4	4.6 (0.1–365.1)	0.3
1st trimester bleeding	7.1 (113)	3.1–13.5	12 (25)	2.6–31.2	1.8 (0.3–8.2)	0.4
2nd and 3rd trimester bleeding	1.8 (112)	0.2–6.3	NR	-	-	1.0
PROM ≤ 37 weeks	0.9 (111)	0.0–4.9	4.0 (25)	0.1–20.4	4.5 (0.1–361.8)	0.3
**Delivery outcomes**						
Weeks at delivery	39.5 (175)	39.2–39.7	39.2 (36)	38.6–39.9	-	0.6
Caesarean section %	33.3 (114)	24.8–42.8	32.0 (25)	15.0–53.5	1.1 (0.4–3.1)	1.0
Induced vaginal labour (%)	9.6 (73)	3.9–18.8	18.8 (16)	4.1–45.7	2.2 (0.3–11.1)	0.4
Puerperal problems	2.7 (112)	0.6–7.6	NR	-	-	-
Preterm births (≤37weeks)	6.3 (175)	3.2–11.0	2.8 (36)	0.1–14.5	2.3 (0.3–103.6)	0.7
Very preterm births (≤34weeks)	2.9 (175)	0.9–6.5	2.8 (36)	0.1–14.5	1.0 (0.1–50.1)	1.0
**Neonatal outcomes**						
Neonatal sex %	-	-	-	-	-	-
Female neonates	49.1 (171)	41.4–56.9	32.4 (34)	17.4–50.5	2.0 (0.9–4.9)	0.1
Male neonates	50.9 (171)	43.1–58.6	67.7 (34)	49.5–82.6		
Birth weight (kg)	3.21 (112)	3.1.5–3.31	3.20 (17)	2.82–3.50	-	0.8
Low birth weight (≤2500 g)	7.1 (112)	3.1–13.6	11.8 (17)	1.5–36.4	1.7 (0.2–9.9)	0.6
Very low birth weight (≤1500 g)	0.9 (112)	0.0–4.9	5.9 (17)	0.2–28.7	6.8 (0.1–546.6)	0.3
Birth height (cm)	50.1 (145)	49.7–50.5	49.6 (30)	48.5–50.6	-	0.3
Birth head circumference	35.2 (92)	35.0–35.5	34.7 (23)	33.6–35.8	-	0.3
Apgar score at 1 min	8.7 (102)	8.5–8.9	8.5 (20)	8.0–8.9	-	0.3
Apgar score at 5 min	9.7 (97)	9.5–9.8	9.7 (20)	9.5–9.9	-	0.8
Apgar score at 10 min	9.8 (8)	9.3–10.2	NR	-	-	-
Admission to NICU %	5.4 (167)	2.5–10.0	6.1 (33)	0.7–20.2	1.1 (0.1–5.9)	1.0

Note: Values are expressed as mean or proportions (with its sample size). SDF—sperm DNA fragmentation; OR—odds ratio; CI—confidence interval; Hb—hemoglobin; PROM—premature rupture of membranes; NR—not reported; NICU—neonatal intensive care unit.

**Table 3:** Obstetric and perinatal outcomes in singleton pregnancies of couples using donated oocytes according to SDF value (*n* = 212).

Variables	≤15% SDF (95%CI)		>15% SDF (95%CI)		OR (95% CI)	*p*-Value
**Pregnancy outcomes**						
Gestational diabetes %	2.9 (136)	0.8–7.4	12.5 (16)	1.6–38.4	4.6 (0.4–35.9)	0.1
Anaemia (Hb ≤ 11 g/dL) %	1.5 (136)	0.2–5.2	-	-	-	1.0
Pre-eclampsia %	2.2 (134)	0.5–6.4	0	-	-	1.0
Threatened preterm labour %	2.9 (136)	0.8–7.4	1 (6.3%)	0.2–30.2	2.2 (0.0–24.1)	0.4
1st trimester bleeding	13.2 (136)	8.0–20.1	25.0 (16)	7.3–52.4	2.2 (0.5–8.3)	0.3
2nd and 3rd trimester bleeding	3.7 (136)	1.2–8.4	6.3 (16)	0.2–30.2	1.7 (0.0–17.1)	0.5
PROM ≤ 37 weeks	2.2 (135)	0.5–6.4	12.5 (16)	1.6–38.4	6.2 (0.5–58.7)	0.1
**Delivery outcomes**						
Weeks at delivery	38.8 (181)	38.5–39.2	39.0 (31)	38.2–39.8	-	0.8
Caesarean section %	53.7 (136)	44.9–62.3	43.8 (16)	19.8–70.1	1.5 (0.5–5.0)	0.6
Induced vaginal labour (%)	9.4 (64)	3.5–19.3	44.4 (9)	13.7–78.8	7.4 (1.2–46.7)	0.02
Puerperal problems	NR	-	NR	-	-	-
Preterm births (≤37 weeks)	10.5 (181)	6.4–15.9	16.1 (31)	5.5–33.7	0.6 (0.2–2.3)	0.4
Very preterm births (≤34 weeks)	3.3 (181)	1.2–7.1	6.5 (31)	(0.8–21.4)	0.5 (0.1–5.3)	0.3
**Neonatal outcomes**						
Neonatal sex %	-	-	-	-	-	
Female neonates	53.4 (178)	45.8–60.9	48.4 (31)	30.2–66.9	1.2 (0.5–2.8)	0.7
Male neonates	46.6 (178)	39.1–54.2	51.6 (31)	33.1–69.9		
Birth weight (kg)	3.10 (122)	3.00–3.20	2.89 (17)	2.62–3.15	-	0.2
Low birth weight (≤2500 g)	9.0 (122)	4.6–15.6	17.7 (17)	3.8–43.4	2.2 (0.3–9.6)	0.4
Very low birth weight (≤1500 g)	0.8 (122)	0.0–4.5	NR	-	-	0.2
Birth height (cm)	49.7 (139)	49.3–50.1	49.6 (21)	48.6–50.6	-	0.9
Birth head circumference	34.7 (94)	34.4–35.1	34.7 (12)	33.8–35.5	-	0.9
Apgar score at 1 min	8.6 (92)	8.3–8.8	8.2 (13)	7.5–8.8	-	0.3
Apgar score at 5 min	9.7 (89)	9.5–9.8	9.6 (11)	9.3–9.9	-	0.9
Apgar score at 10 min	9.6 (7)	9.0–10.2	NR	-	-	-
Admission to NICU %	11.9 (168)	7.4–17.8	10.7 (28)	2.3–28.2	0.9 (0.2–3.3)	1.0

Note: Values are expressed as mean or proportions (with its sample size). SDF—sperm DNA fragmentation; OR—odds ratio; CI—confidence interval; Hb—haemoglobin; PROM—premature rupture of membranes; NR—not reported; NICU—neonatal intensive care unit.

**Table 4:** Logistic regression analysis for singleton pregnancy cycles of preterm birth (≤37 weeks), very preterm birth (≤34 weeks), female neonates, and low birth weight (≤2500 g) by high (>15%) SDF level.

	Autologous Oocytes		Donated Oocytes	
Outcome Variable	OR (95% CI)	*p*-Value	OR (95% CI)	*p*-Value
Preterm birth (≤37 weeks)	0.43 (0.0–2.3) *n* = 36	0.42	1.64 (0.5–4.5) *n* = 31	0.36
Preterm birth (≤37 weeks) (adjusted)	0.31 (0.0–2.2) *n* = 6	0.33	1.76 (0.5–5.9) *n* = 8	0.40
Very preterm birth (≤34 weeks)	0.97 (0.0–6.3) *n* = 36	0.98	2.0 (0.3–9.2) *n* = 31	0.41
Very preterm birth (≤34 weeks) (adjusted)	0.80 (0.0–6.7) *n* = 6	0.86	2.6 (0.4–14.3) *n* = 8	0.28
Female neonates	0.50 (0.2–1.1) *n* = 31	0.08	0.82 (0.4–1.8) *n* = 31	0.61
Female neonates (adjusted)	0.35 (0.1–0.9) *n* = 6	0.04	0.70 (0.3–1.6) *n* = 8	0.39
Birth weight ≤ 2500 g	1.7 (0.3–7.8) *n* = 17	0.51	2.20 (0.5–8.0) *n* = 17	0.28
Birth weight ≤ 2500 g (adjusted)	1.6 (0.2–9.4) *n* = 6	0.64	2.42 (0.3–13.2) *n* = 8	0.33

The variables female and male age, female body mass index (BMI), day of embryo transfer (D3 or D5), and endometrial thickness were included in the analysis as potential confounders. AOR—adjusted odds ratio.

## Data Availability

The data presented in this study are available on request from the corresponding author. The data are not publicly available due to privacy.

## Supplementary Materials

The following supporting information can be downloaded at: https://www.mdpi.com/article/10.3390/jcm12216802/s1, Table S1—Baseline demographics and characteristics of IVF cycles of couples using autologous and donated oocytes according to SDF value in multiple deliveries; Table S2—Obstetric and perinatal outcomes in multiple deliveries of couples using autologous oocytes according to SDF value; Table S3—Obstetric and perinatal outcomes in multiple deliveries of couples using donated oocytes according to SDF value.
