# Supplementary material for: Elevated Sperm DNA Damage in IVF–ICSI Treatments Is Not Related to Pregnancy Complications and Adverse Neonatal Outcomes
Source: J Clin Med. 2023 Oct 27;12(21):6802. doi: 10.3390/jcm12216802 (PMC10649005; doi:10.3390/jcm12216802)
Supplement: Supplementary file 1 [file jcm-12-06802-s001.zip › Supplemental Table S1-demographics multiple deliveries.pdf]

**Supplemental Table S1**. Baseline demographics and characteristics of IVF cycles of couples using autologous and donated oocytes according to SDF value in multiple deliveries.

| | Autologous oocytes | | | | | Donated oocytes | | | | |
|---|---|---|---|---|---|---|---|---|---|---|
| | ≤15% SDF (95%CI) | | >15% SDF (95%CI) | | P-value | ≤15% SDF (95%CI) | | ≤15% SDF (95%CI) | | P-value |
| *Variables* | | | | | | | | | | |
| **Maternal age (years)** | 35.3 (12) | 33.9-36.8 | 35.3 (4) | 32.6-38.4 | 0.9 | 39.1 (19) | 37.5-40.7 | 39.8 (4) | 38.3-41.2 | 0.6 |
| **Female BMI (kg/m2)** | 23.1 (21) | 21.3-24.9 | 23.5 (4) | 19.4-27.7 | 0.9 | 23.0 (27) | 21.5-24.5 | 27.4 (3) | 19.1-35.6 | 0.4 |
| **Male age (years)** | 36.8 (25) | 35.2-38.4 | 36.3 (6) | 33.9-38.8 | 0.8 | 39.3 (29) | 37.6-41.1 | 38.5 (4) | 34.5-42.5 | 0.7 |
| **Male BMI (kg/m2)** | 22.9 (23) | 21.2-24.6 | 22.7 (5) | 19.2-26.3) | 0.9 | 23.0 (29) | 21.6-24.4 | 25.3 (4) | 20.2-30.4 | 0.5 |
| **SDF (%)** | 6 (25) | 0.0-0.1 | 21 (6) | 15.0-26.0 | ≤0.001 | 6.0 (29) | 5.0-7.0 | 22.0 (4) | 16.0-27.0 | 0.01 |
| **Previous miscarriages (>=1)** | 1.5 (4) | 0.9-2.1 | - | - | - | 2 (8) | 1.4-2.6 | 4 (1) | - | - |
| **Parous women (>=1)** | 1 (8.3%) | 0.2-38.5 | - | - | - | 10.5 (19) | 1.3-33.1 | - | - | - |
| **Previous preterm deliveries (≤=1)** | 0 | - | 0 | - | - | - | - | - | - | - |
| **Previous medical disorders** | 3 (25%) | 5.5-57.2 | 1 (25%) | 0.6-80.6 | 1.0 | 31.6 (19) | 12.6-56.6 | 25.0 (4) | 0.6-80.6 | 1.0 |
| **Days of stimulation** | 10.4 (26) | 9.7-11.0 | 9.5 (6) | 8.5-10.5 | 0.2 | 10.7 (29) | 10.1-11.2 | 10.0 (4) | 10.0-10.0 | 0.03 |
| **FSH total dose (IU)** | 1584.4 (24) | 1334.1-1834.7 | 1625.0 (4) | 1069.6-2180.4 | 0.9 | 1681.5 (27) | 1500.2-1862.7 | 1412.5 (4) | 1255.6-1569.4 | 0.05 |
| **E2 on day of hCG (pg/mL)** | 2039 (26) | 1689.8-2388.2 | 2055.8 (6) | 1119.4-2992.3 | 1.0 | 2524.0 (28) | 2062.8-2985.1 | 3748.5 (4) | 1060.9-6436.1 | 0.4 |
| **P4 on day of hCG (pg/mL)** | 0.7 (25) | 0.6-0.9 | 0.6 (6) | 0.5-0.7 | 0.1 | 0.5 (1) | - | - | - | - |
| **Last endometrial thickness** | 10.0 (24) | 9.2-10.8 | 10.0 (6) | 9.1-10.9 | 0.9 | 9.3 (26) | 8.7-9.9 | 9.9 (4) | 9.0-10.8 | 0.3 |
| **Number of oocytes retrieved** | 12.6 (26) | 10.6-14.7 | 10.8 (6) | 6.7-14.9 | 0.5 | 22.8 (29) | 18.8-26.8 | 19.5 (4) | 10.7-28.3 | 0.5 |
| **Number of inseminations/patients** | 1.0 (25) | 1.0-1.0 | 1.0 (6) | 1.0-1.0 | - | 1.0 (29) | - | 1 | - | - |
| **Embryos transferred/ patient** | 1.4 (25) | 1.2-1.6 | 1.3 (6) | 0.9-1.8 | 0.8 | 1.8 (29) | 1.6-1.9 | 1.5 (4) | 0.9-2.1 | 0.4 |
| **Day-3 ET** | 2 | - | 2 | - | - | 1 | - | 0 | - | - |
| **Blastocyst ET** | 23 | - | 4 | - | - | 28 | - | 4 | - | - |

Note: : Values are expressed as mean or proportions (with its sample size).
SDF: sperm DNA fragmentation; CI: confidence interval; BMI: body mass index; FSH: follicle stimulating hormone; LH: luteinizing hormone; E2: estradiol; P4: progesterone; hCG: human chorionic gonadotropin; ET: embryo transfer.
