# Supplementary material for: Elevated Sperm DNA Damage in IVF–ICSI Treatments Is Not Related to Pregnancy Complications and Adverse Neonatal Outcomes
Source: J Clin Med. 2023 Oct 27;12(21):6802. doi: 10.3390/jcm12216802 (PMC10649005; doi:10.3390/jcm12216802)
Supplement: Supplementary file 1 [file jcm-12-06802-s001.zip › Supplementary Table S2-clinical outcomes multiple own.pdf]

Supplementary Table S2. Obstetric and perinatal outcomes in multiple deliveries of couples using autologous oocytes according to SDF value (n=31).

Variables	≤15% SDF (95%CI)		SDF>15% SDF (95%CI)		OR (95% IC)	P-value
**Pregnancy outcomes**						
Gestational diabetes	8.3 (12)	0.2-38.5	-	-		-
Anaemia (Hb ≤11 g/dL)	8.3 (12)	0.2-38.5	-	-		1.0
Pre-eclampsia	8.3 (12)	0.2-38.5	-	-		-
Threatened preterm labour	25.0 (12)	5.5-57.2	25.0 (4)	0.6-80.6		1.0
1st trimester bleeding	41.7 (12)	15.2-72.3	-	-		1.0
2nd and 3rd trimester bleeding	16.7 (12)	2.1-48.4	25.0 (4)	0.6-80.6		1.0
PROM ≤37 weeks	0	-	0	-		-
**Delivery outcomes**						
Weeks at delivery	36.3 (25)	35.6-37.0	37.7 (6)	36.1-39.4		0.1
Caesarean section	83.3 (12)	51.6-97.9	75.0 (4)	19.4-99.4		1.0
Induced vaginal labour	50 (2)	1.3-98.7	-	-		-
Puerperal problems	8.3 (12)	0.2-38.5	-	-		-
Preterm births (≤37 weeks)	56.0 (25)	34.9-75.6	16.7 (6)	0.4-64.1	6.0 (0.6-321.4)	0.2
Very preterm births (≤34 weeks)	3.0 (25)	2.6-31.2	NR	-	-	-
**Neonatal outcomes**						
Female neonates	51.1 (47)	36.1-65.9	41.7 (12)	15.2-72.3	1.5 (0.3-6.7)	0.8
Male neonates	48.9 (47)	34.1-63.9	58.3 (12)	27.7-84.3		
Birth weight (kg)	2.4(36)	2.3-2.5	2.7(10)	2.6-2.8		**0.01**
Low birth weight (≤2,500 g)	58.3 (36)	40.8-74.5	10.0 (10)	0.3-44.5	0.1 (0.0-0.7)	**0.01**
Very low birth weight (≤1500 g)	NR		NR		-	-
Birth height (cm)	47.0 (32)	46.4-47.5	48.0 (10)	46.2-49.7		0.3
Birth head circumference	33.1 (16)	32.7-33.5	33.0 (6)	31.9-34.1		0.8
Apgar score at 1 min	8.5 (20)	8.2-8.8	8.3 (10)	7.7-8.9		0.6
Apgar score at 5 min	9.3 (18)	9.1-9.6	9.3 (10)	8.9-9.7		0.9
Apgar score at 10 min	10 (1)	-	-	-	-	-
Admission to NICU	27.3 (44)	15.0-42.8	8.3 (12)	0.2-38.5	0.3 (0.0-2.1)	0.3

Note: Values are expressed as mean or proportions (with its sample size).
SDF: sperm DNA fragmentation; OR: odds ratio; CI: confidence interval; Hb: haemoglobin; PROM: premature rupture of membranes; NR: not reported; NICU: neonatal intensive care unit.
