# Supplementary material for: Elevated Sperm DNA Damage in IVF–ICSI Treatments Is Not Related to Pregnancy Complications and Adverse Neonatal Outcomes
Source: J Clin Med. 2023 Oct 27;12(21):6802. doi: 10.3390/jcm12216802 (PMC10649005; doi:10.3390/jcm12216802)
Supplement: Supplementary file 1 [file jcm-12-06802-s001.zip › Supplementary Table S3-clinical outcomes multiple donated.pdf]

Supplementary Table S3. Obstetric and perinatal outcomes in multiple deliveries of couples using donated oocytes according to SDF value (n=33).

Variables	≤15% SDF (95%CI)		>15% SDF (95%CI)		OR (95% IC)	P-value
Pregnancy outcomes						
Gestational diabetes	21.1 (19)	6.1-45.6	25.0 (4)	0.6-80.6		1
Anaemia (Hb ≤11 g/dL)	0 (19)	82.4-100	0 (4)	39.8-100		-
Pre-eclampsia	15.8 (19)	3.4-39.6	-	-		-
Threatened preterm labour	15.8 (19)	3.4-39.6	-	-		-
1st trimester bleeding	47.4 (9)	24.5-71.1	-	-		-
2nd and 3rd trimester bleeding	-	-	-	-		-
PROM ≤37 weeks	21.1 (19)	6.1-45.6	50.0 (4)	6.8-93.2		0,3
Delivery outcomes						
Weeks at delivery	35.9 (29)	34.7-37.2	36.1 (4)	34.7-37.4	-	0.9
Caesarean section	57.9 (19)	33.5-79.8	100 (4)	39.8-100.0		1
Induced vaginal labour	50.0 (4)	15.7-84.3	-	-		-
Puerperal problems	15.8 (19)	3.4-29.6	-	-		-
Preterm births (≤37 weeks)	48.3 (29)	29.5-67.5	75.0 (4)	19.4-99.4	0.3 (0.0-4.6)	0.6
Very preterm births (≤34 weeks)	13.8 (29)	3.9-31.7	NR	-	-	-
Neonatal outcomes						
Female neonates	48.3 (58)	35.0-61.8	50.0 (8)	15.7-84.3	0.9 (0.2-5.5)	1.0
Male neonates	51.7 (58)	38.2-65.1	50.0 (8)	15.7-84.3		
Birth weight (kg)	2.2 (54)	2.1-2.4	2.4 (8)	2.2-2.6		0.1
Low birth weight (≤2,500 g)	55.6 (54)	41.4-69.1	62.5 (8)	24.5-91.5	1.3 (0.2-9.4)	1.0
Very low birth weight (≤1500 g)	13.0 (54)	5.4-24.9	NR	-		0.6
Birth height (cm)	46.1 (34)	45.2-47.2	47.0 (6)	46.1-47.1		0.2
Birth head circumference	32.5 (12)	31.3-33.7	33.0 (4)	31.6-34.4		0.6
Apgar score at 1 min	8.6 (18)	8.1-9.0	7.5 (4)	6.2-8.8		0.2
Apgar score at 5 min	9.4 (16)	8.9-9.8	9.5 (4)	8.9-10.1		0.7
Apgar score at 10 min		-	NR	-		-
Admission to NICU	42.9 (56)	29.7-56.8	12.5 (8)	0.3-52.7	0.2 (0.0-1.7)	0.1

Note: Values are expressed as mean or proportions (with its sample size).
SDF: sperm DNA fragmentation; OR: odds ratio; CI: confidence interval; Hb: haemoglobin; PROM: premature rupture of membranes; NR: not reported; NICU: neonatal intensive care unit.
